# Distinct effects of slow and fast theta tACS in enhancing temporal memory

**DOI:** 10.1162/imag_a_00332

**Published:** 2024-10-24

**Authors:** Yuejuan Wang, Peter De Weerd, Alexander T. Sack, Vincent van de Ven

**Affiliations:** Department of Cognitive Neuroscience, Faculty of Psychology and Neuroscience, Maastricht University, Maastricht, The Netherlands

**Keywords:** transcranial alternating current stimulation, temporal memory, event segmentation, slow theta oscillations

## Abstract

Temporal memory plays a crucial role in organizing real-life events into meaningful segments. Previous research suggests that the clustering of temporally related information builds on the neural correlates of associative memory, including theta oscillations. Transcranial alternating current stimulation (tACS) provides a means of modulating theta oscillations within associative memory networks, possibly including hippocampal modulation when targeting the parietal cortex. Theta oscillations are not limited to a single frequency range, but instead, exhibit functional specialization, with slow theta (3 Hz) implicated in short-term episodic memory formation and fast theta (8 Hz) involved in spatial navigation. Our study aimed to investigate the distinct effects of slow and fast theta stimulation on temporal memory. Participants encoded visual objects paired with frame color while receiving tACS stimulation at 3 Hz, 8 Hz, or sham targeting the left parietal cortex. The frame color would change after every eight objects, establishing a context boundary with each color change. Subsequently, participants performed a timeline task to assess temporal memory performance. Results showed that slow, but not fast, theta stimulation led to an enhancement in temporal accuracy (absolute temporal error) compared to sham tACS, in support of our main hypothesis. Under sham stimulation, participants consistently underestimated the temporal position of items presented further away from boundary, compared to those presented at boundary. This finding resembled temporal compression observed during event segmentation. Interestingly, fast, but not slow, theta stimulation reduced this temporal bias (rated position–actual position). This study represents the first tACS evidence for differential contributions of slow versus fast theta to temporal memory formation in humans. We speculate that parietal theta tACS may modulate the hippocampus and facilitate temporal memory formation.

## 1 Introduction

Temporal memory is the ability to retrieve the temporal context of a unique event from episodic memory. Many researchers have in fact suggested that recollection of temporal context is an integral feature of episodic memory (Eichenbaum, 2013;Friedman, 2004;Tulving, 1984). There is ample evidence that neural oscillatory activity, particularly in the theta range of 3 to 8 Hz, plays an important role in episodic memory (Berens & Horner, 2017;Clouter et al., 2017;Herweg et al., 2020), suggesting that theta oscillations also support temporal memory formation. A few studies (For a review,Herweg et al., 2020) have, indeed, reported such correlations between theta oscillatory power and temporal memory performance, but the functional relevance of these theta oscillations in the context of temporal memory remains unclear. In the current study, we used transcranial alternating current stimulation (tACS) to experimentally entrain both slow and fast theta oscillatory activity to experimentally assess the role of oscillatory stimulation in temporal memory performance.

When encoding a series of experiences, we tend to segment those experiences into discrete temporal clusters in memory, based on perceived changes in contextual features of the experiences (Brunec et al., 2018;Clewett & Davachi, 2017;Zacks, 2020). The contextual changes may thus serve as boundaries separating the contexts of different event models in memory, which benefit memory formation and recognition (Flores et al., 2017;Huff et al., 2014;Pettijohn et al., 2016). Boundaries act as a scaffold by which event memory is shaped. For example, some studies demonstrated that participants took longer to encode items encountered at boundaries, compared to items presented away from those boundaries (Heusser et al., 2018;van de Ven et al., 2023;Wen & Egner, 2022). In recognition memory tests following sequential item presentations, boundary items were better recognized afterwards than items that were presented away from those boundaries (Huff et al., 2018;Swallow et al., 2009). This suggests that contextual changes transiently increase attentional or working memory processing, thereby facilitating memory formation for items at the boundary. Previous studies have also provided evidence that boundaries play a role in temporal memory (DuBrow & Davachi, 2014;Ezzyat & Davachi, 2014;Heusser et al., 2018;van de Ven et al., 2022,^2023^). For example, in the study byHeusser et al. (2018), participants encoded a series of visual objects presented within a frame that changed color after every sixth item to define contextual boundaries. Results showed that temporal order memory (which of two test items was encoded first) was superior for items that were encoded in the same context compared to items drawn from different color contexts. The researchers suggested that the items perceived in the same context underwent stronger associative encoding compared to the items segmented in adjacent contexts (DuBrow & Davachi, 2014;Ezzyat & Davachi, 2011;Heusser et al., 2018). In addition, they proposed that non-boundary items encoded further away from boundaries suffered from weaker memory accuracy, rendering subsequent memory retrieval more difficult. When subsequently retrieving the temporal context of boundary-segmented events, participants would remember the segments as having lasted shorter than they actually did, resulting in a reported temporal compression due to the forgetting of “time slices” within an event away from the boundary (D’Argembeau et al., 2022;Jeunehomme et al., 2018;Jeunehomme & D’Argembeau, 2020). Boundaries could thus affect the temporal structure of experience by facilitating temporal memory accuracy for boundary items and by inducing temporal compression of non-boundary items.

One way in which boundaries could affect temporal memory is by modulating oscillatory brain activity underlying memory formation. There is ample evidence that neural oscillations in the theta range of 3–8 Hz support episodic memory formation (Berens & Horner, 2017;Herweg et al., 2020;Lega et al., 2012). The role of theta oscillations in the formation of temporal memory has been relatively underexplored, but recent research has presented supporting evidence for this proposition. For example, theta power measured with EEG during working memory maintenance of temporal order was enhanced compared to maintenance of visual item information (Hsieh et al., 2011) or spatial location (Roberts et al., 2013). In another study (Crivelli-Decker et al., 2018), participants learned sequences of visual objects that were repeated in either a fixed or a randomized order across repetitions. For each object, participants provided a semantic categorization response. Results showed that post-response theta power decreased for progressively later presented objects in a sequence when the order was fixed across repetitions, compared to when the order was randomized, suggesting that theta oscillations contributed to temporal sequence learning.

There is further evidence that theta-gamma phase coupling may play a role in temporal segmentation. It has previously been suggested that associative binding in memory is brought about by coupling of gamma bursts for discrete sensory inputs to discrete phase parts of the ongoing theta cycle (Griffiths & Fuentemilla, 2020;Jensen, 2006;Lisman & Idiart, 1995;Lisman & Jensen, 2013), with the number of gamma bursts to non-overlapping phase parts limiting the capacity of items held in short-term memory. This model was extended to temporal memory in a magnetoencephalography (MEG) study (Heusser et al., 2016), in which they showed that sequential phase-locking of gamma bursts within the theta cycle for discrete visual items during encoding predicted subsequent temporal order memory accuracy of those items, suggesting that theta-gamma phase locking could serve as the “temporal glue” by which items are bound in memory. Research in rodents further suggests that temporal dynamics of the theta-gamma coupling may support temporal segmentation of phase-locked clusters of gamma bursts into different sequences of theta cycles, and that phase-locking correlated with the animal’s location relative to spatial boundaries (Griffiths & Fuentemilla, 2020;Gupta et al., 2012;McKenzie & Buzsáki, 2016). Further,Gupta et al. (2012)showed that phase-locked gamma activity within a theta cycle was biased towards coding locations ahead of the animal’s current location at the start of a spatial boundary and tended to code locations behind the animal when being close to the next boundary in the trajectory, thereby providing a temporal representation of spatial boundaries in the environment.

Finally, slower oscillations within the theta band may contribute more to segmentation and temporal memory than faster oscillations. Auxiliary results byGupta et al. (2012)’s study of spatial segmentation and theta-gamma coupling also showed that longer boundary-based spatial segments were correlated with lower theta frequency and increased number of phase-locked gamma bursts, suggesting that slower theta frequencies may allow coupling of more items in longer lasting events. In addition, human studies have shown that within the theta range, there may be a functional mnemonic specialization for different frequency ranges (Goyal et al., 2020;Kota et al., 2020;Lega et al., 2012). Intracranial hippocampal recordings in human brain surgery patients have shown that 3 Hz oscillatory power (slow theta) was correlated with memory encoding (Lega et al., 2012), while 8 Hz (fast theta) was associated with spatial navigation, but not mnemonic processing (Goyal et al., 2020). This notion of functionally dissociated theta rhythms was further supported by a tACS study that administered slow (4 Hz) and fast (7 Hz) theta stimulation over parietal cortex (Wolinski et al., 2018), showing that slow theta stimulation facilitated visuospatial working memory capacity compared to sham stimulation while fast theta stimulation impaired working memory capacity (Wolinski et al., 2018). Taken together, these studies thus suggest that slow but not fast theta oscillations are involved in temporal memory formation.

However, the results of these studies are largely correlational, which prevents assessment of the causal or at least functional role of oscillations in temporal memory. A recent review about modulating human memory via the entrainment of brain oscillations proposed that memory processes can be altered when brain oscillations are being interfered with (Hanslmayr et al., 2019). Transcranial alternating current stimulation (tACS) is a form of non-invasive brain stimulation that permits experimental modulation of ongoing brain oscillations to affect respective cognitive processes. Several tACS studies (Helfrich et al., 2014;Herrmann et al., 2013;Meng et al., 2021;Vulić et al., 2021;Živanović et al., 2022), indeed, provided empirical evidence indicating that theta tACS can affect neural processing of the associative memory system. For example, individual theta tACS (Živanović et al., 2022) or theta (5 Hz) oscillatory transcranial direct current stimulation (Vulić et al., 2021;Živanović et al., 2022) delivered to left parietal cortex during a short-term task improved performance compared to sham stimulation. In another study,Meng et al. (2021)administered 6 Hz or sham tACS over left parietal lobe (P3) while participants encoded a sequence of faces paired with scenes, and then completed an associative memory and a visual recognition test after stimulation ended. Compared to sham stimulation, 6 Hz stimulation resulted in decreased associative memory performance, but did not affect visual recognition performance for individual items. Furthermore, scalp-based tACS may indirectly modulate hippocampal activity, particularly during cognitive states of enhanced hippocampal-cortical connectivity (Krause et al., 2019;Vieira et al., 2020;Wischnewski et al., 2023). This may be particularly relevant when stimulating over inferior parietal cortex, which shows strong functional connectivity with hippocampal structures during resting states and memory functions (van de Ven et al., 2013;Vincent et al., 2006;Wang et al., 2014).

In the current study, we therefore tested the contribution of slow (3 Hz) and fast (8 Hz) theta oscillations (sham stimulation as the control condition) in the encoding of temporal structure in memory using tACS administered over the left parietal lobe (P3). During the task, participants encoded visual objects that were presented within a colored frame while undergoing different tACS conditions (3 Hz, 8 Hz, and sham) for 5 minutes. After encoding, they were asked to indicate when (i.e., at which sequential position) a picture was presented during the encoding phase on a visual analog scale (VAS) that represented the timeline of the sequence. We hypothesized that 3 Hz stimulation, but not 8 Hz stimulation, would enhance temporal position memory accuracy, in line with the suggested functional specialization of slow theta in episodic memory formation.

## 2 Method

### 2.1 Participants

We recruited participants for a counterbalanced within-subject design. With a conservatively estimated effect size*f*= 0.30 (*alpha*= 0.05,*power*= 0.8), a power analysis for a one-factor three-level within-subject design using G*Power indicated a minimum sample size of 20 participants. We initially recruited 24 undergraduate students for the experiment, based on a multiple of 6 to accommodate counterbalancing (see below). The data of one participant were discarded due to the high frequency (53%) with which the subject selected the same VAS location in the timeline task (see procedures below) and was replaced by a 25th participant that completed the same tACS order as the discarded participant. Participants (mean age = 19.84, 20 females) were recruited via an online recruitment system of Maastricht University. All participants were prescreened for their eligibility and safety to undergo transcranial electric stimulation. Written informed consent was obtained from each participant before starting the experiment. Participants received either monetary compensation or course credits. All study procedures were approved by the ethics review committee of psychology and neuroscience (ERCPN) from the Faculty of Psychology and Neuroscience (FPN) at Maastricht University.

### 2.2 Task procedure

Each participant completed three consecutive tACS sessions in one experiment sitting. In each tACS session, participants completed three tasks: an encoding task, a distractor task, and a temporal memory task, with one of three tACS frequencies (3 Hz, 8 Hz, or sham) administered during the encoding task. All tasks were programmed in PsychoPy 3 (Peirce et al., 2019). The order of the three tACS conditions was counterbalanced across participants, with each of the six unique orders repeated four times across the participants.

**Fig. 1.**
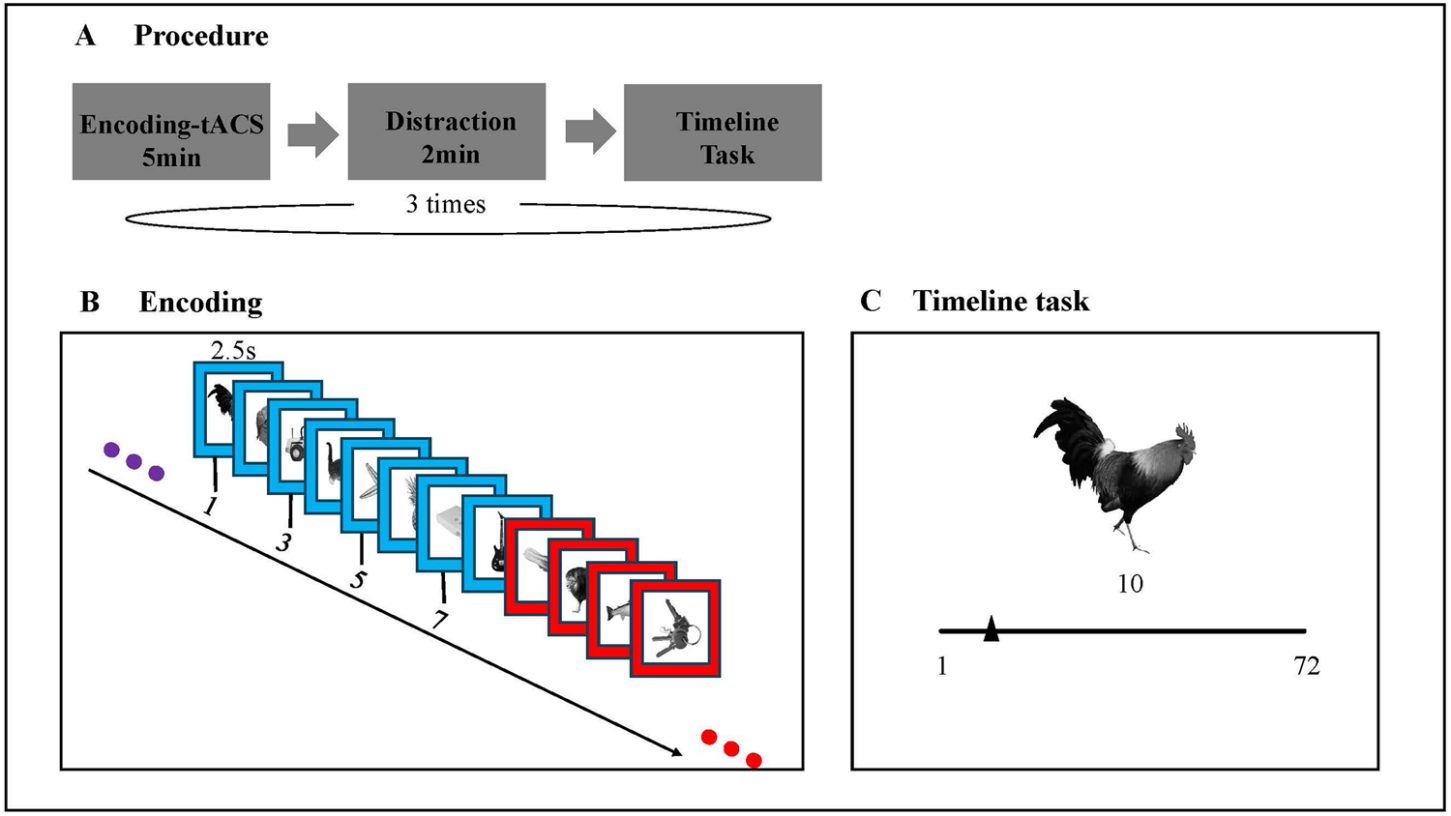
Experimental design. (A) Participants performed encoding task, distraction task and timeline task. This procedure was repeated three times with different order of three tACS conditions among participants during encoding. (B) Participants indicated how pleasant they considered the combination of the object and color frame during encoding on a 4-point scale. (C) In timeline task, participants dragged the slider to indicate the position of the image in the previous encoding phase. The objects from the 1st, 3rd, 5th, and 7th position from each color context were tested.

During the encoding task of each session, participants viewed 72 gray scale pictures of various objects (Fig. 1B). Pictures were selected from an online image database (Kovalenko et al., 2012). For each participant, the software randomly selected 216 images from a larger set of 371 images and allocated them across the three encoding sessions. Each picture was presented within a colored picture frame, which changed color after every eight pictures. This resulted in a total of nine different frame colors for each encoding session, with each color randomly drawn from an RGB color set without repetition. At the beginning of each trial, participants saw a picture placed in the middle of the screen for 2.5 s and had to indicate how pleasant they found the combination of the picture with the frame color for drawing attention to the picture and the frame color. Participants indicated their judgment on a four-item rating scale (1 “very unpleasant” up to 4 “very pleasant”) by pressing corresponding keys on the computer keyboard with their dominant hand. Participants had to respond within the 2.5 s in which the picture was presented, and response time was logged for offline analysis. Every picture was followed by an inter-stimulus interval that lasted 2 s in which a fixation cross was presented. Each encoding phase lasted approximately 5 minutes.

After the encoding task, participants completed a simple arithmetic task (for example: 19 − 13 + 4 = 10, K - “True”, L - “False”) for 2 minutes, which served as a distraction task to suppress memory rehearsal. The distraction task was followed by a timeline task, in which participants had to recall the position (ranging from 1 to 72) of a test item relative to the sequence presented during the encoding phase (Fig. 1C). The item was placed in the middle of the screen, and a visual analog scale (VAS) was presented as a representation of the encoding task timeline comprising the 72 positions with tick-marks at the beginning (1) and the end (72). Participants could indicate the position on the timeline scale by moving a slide marker using the computer mouse without a time limit. The trial started without a slide marker, which would only appear once participants started to move their mouse on the VAS. When moving the slide marker, the selected position (in whole numbers) on the VAS timeline was shown on the screen for feedback to the participant. Participants could freely adapt their timeline position without a time limit and pressed the spacebar to accept their final choice. The task consisted of 36 trials, comprising the 1st (referred to as “boundary item”), 3rd, 5th, and 7th image (“non-boundary items”) from each color context.

To familiarize participants with task procedures and experience with transcranial electric stimulation (see below), they completed a short training version of the encoding (18 trials) and temporal memory tasks (4 trials) prior to the main experiment, using pictures that were not shown during the main experiment. After the third tACS condition, participants were asked to indicate which of the three tACS sessions they thought included sham stimulation. The experiment lasted approximately 90 minutes, including the time necessary to attach the electrodes on the participant’s scalp.

### 2.3 tACS parameters

**Fig. 2.**
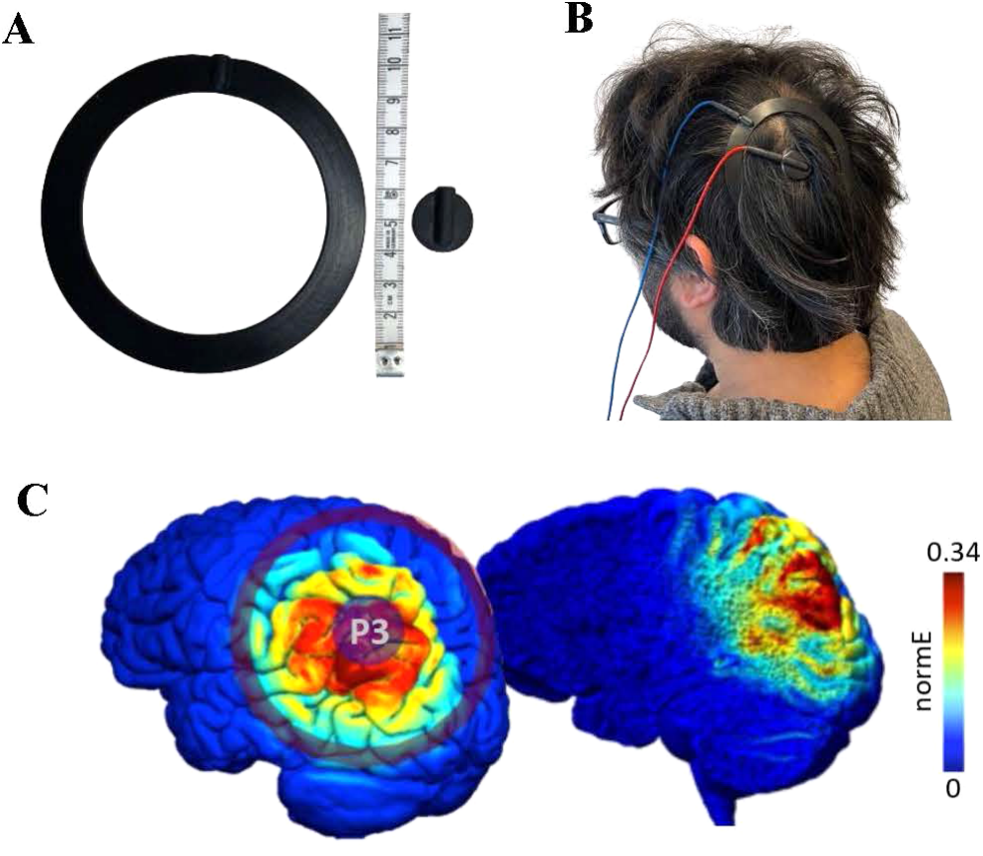
Concentric electrode setup and field simulation. (A) The size of the ring electrodes. The inner electrode is circular and 2 cm in diameter; the outer electrode is ring-shaped with 10 cm diameter between its outer edges and the width is 1.2 cm. (B) The setup of the ring electrodes on the head of a participant. (C) Electric field simulation over the cortical surface. Simulated values are presented in normalized electric field magnitude, V/m.

Electric stimulation was delivered over scalp location P3 (Fig. 2B and C), as identified by the 10–20 EEG electrode positioning system, using concentric electrodes set up that contained an inner and an outer circular electrode (Fig. 2A) for focal stimulation. Simulation of the electric field distribution using SimNIBS (Saturnino et al., 2019) supported a focal stimulation signal of the concentric electrodes (Fig. 2C, see alsoMeng et al. (2021)). To facilitate the conductivity of the electrical signal between electrodes and scalp, adhesive paste was used (Ten 20,https://www.weaverandcompany.com). The impedance was kept below 15 Ω for every participant. We carefully monitored that the paste did not directly connect between the electrodes. After attaching the electrodes and checking the impedance (approximately 30 minutes), participants were familiarized with the sensation of tACS stimulation, during which they were closely monitored for adverse effects or discomfort. For the familiarization, stimulation lasted approximately 1–2 minutes at 5.5 Hz (participants were not made aware of the used stimulation frequency).

tACS was administered with a peak-to-peak intensity of 2 mA. In the slow theta condition, stimulation included 3 Hz with 990 cycles (5.5 minutes × 60 s × 3 Hz). In the fast theta condition, stimulation included 8 Hz with 2640 cycles (5.5 minutes × 60 s × 8 Hz). Finally, in the sham condition, stimulation included 5.5 Hz for 30 s (165 cycles, 0.5 minutes × 60 s × 5.5 Hz). The ramp-up and ramp-down duration was set to 60 cycles (20 s in 3 Hz; 7.5 s in 8 Hz; 10.9 s in sham).

### 2.4 Data analysis

The average response time of the pleasantness judgments about the combination of the picture and frame color during the encoding phase was analyzed using a repeated-measures ANOVA with tACS condition (Sham, 3 Hz, 8 Hz) and encoding position (eight items per segment) as independent variables.

For the timeline task, trials with overly fast (<0.5 s) or slow responses (>15 s) were excluded, which discarded 2.20% of the trials on average across all participants. For each color context, position 1 was considered the “boundary position” and positions 3, 5, and 7 were averaged and dubbed “non-boundary position”. To assess how well participants performed in the task, we compared performance to simulated chance-level accuracy, which follows a non-linear pattern as a function of serial position. We then analyzed two dependent variables: temporal accuracy (the absolute error, i.e., the magnitude of the difference between rated and actual temporal position, irrespective of the direction of the error) and temporal bias (the signed temporal error, i.e., the direction and magnitude of the rated minus actual position). Each dependent variable was averaged across trials for each condition and analyzed using a repeated-measures ANOVA with tACS (Sham, 3 Hz, 8 Hz) and tested within-context position (boundary vs. non-boundary) as independent variables. Significance of posthoc pairwise tests was Bonferroni-corrected for multiple comparisons, with the corrected alpha threshold for three tACS comparisons beingα= 0.05/3 = 0.0167. The results of a 3 (tACS) × 4 (position) ANOVA on response time and temporal error are presented in the Supplementary Materials (see alsoSupplementary Fig. S4).

## 3 Results

### 3.1 Encoding response time

**Fig. 3.**
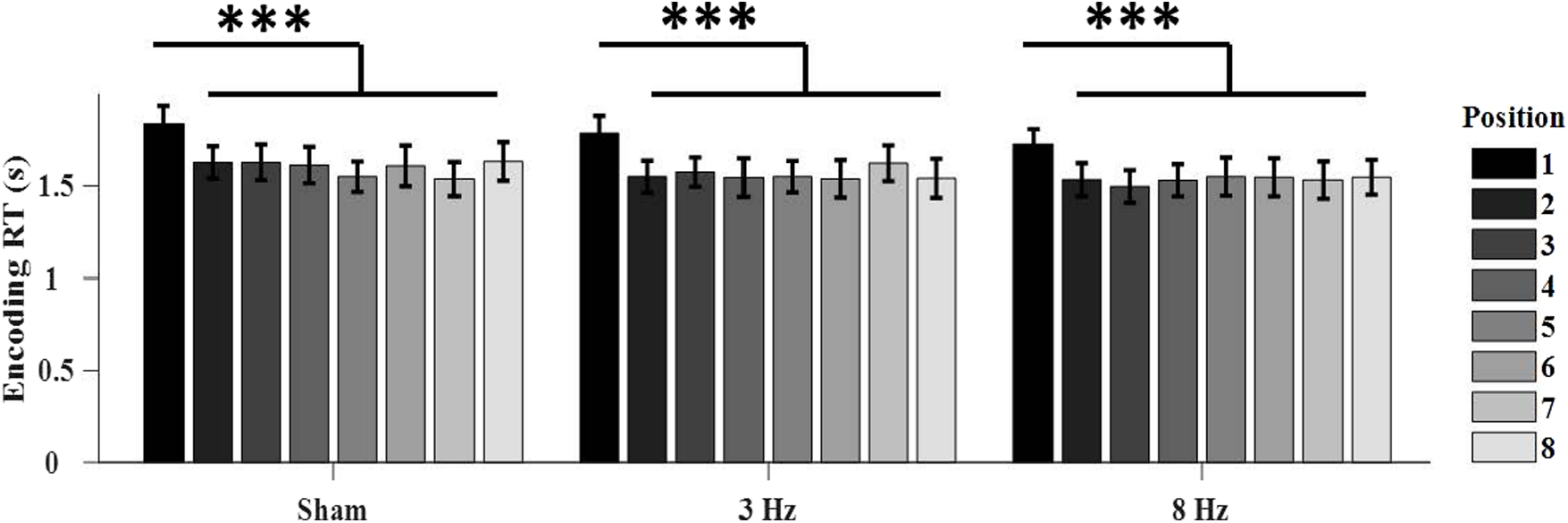
Response time in seconds during encoding as a function of context-based encoding position and tACS condition. Error bars represent standard error of the mean. ***p< 0.001.

The repeated-measures ANOVA revealed a main effect of position (*F*(7, 14) = 18.12,*p*< 0.001,*η_p_^2^*= 0.44) on response time of the pleasantness judgment during encoding (Fig. 3). Participants took longer to respond to boundary items compared to non-boundary items (*ps*< 0.001), which indicated a boundary effect similar to previous behavioral studies (Heusser et al., 2018;van de Ven et al., 2023). The main effect of tACS condition (*p*= 0.121) and the tACS x position interaction effect (*p*= 0.330) were not significant, indicating that tACS frequencies did not alter the boundary effect during the encoding phase.

### 3.2 Temporal position task

#### 3.2.1 Chance performance simulation for temporal errors

To assess chance-level performance for temporal accuracy in the timeline task, we simulated temporal accuracy in the following way. First, we created 10*k*randomly shuffled series of temporal position indexes (from position 1 to 72) to obtain simulated responses. Then, we subtracted from each simulated response the actual position index of that response and obtained the absolute magnitude of the difference to obtain simulated temporal accuracy. Finally, we averaged the simulated accuracies of each position across the 10*k*series. This procedure resulted in a U-shaped curve of simulated temporal accuracy at chance-level, with smaller errors for middle positions and higher errors for starting and ending positions (black curve inFig. 4A). The average simulated absolute temporal error across all positions was 24. We also simulated temporal bias of chance-level performance by averaging the simulated signed errors, which linearly decreased over temporal positions (black curve inFig. 4B). Average temporal bias across all positions was 0.

**Fig. 4.**
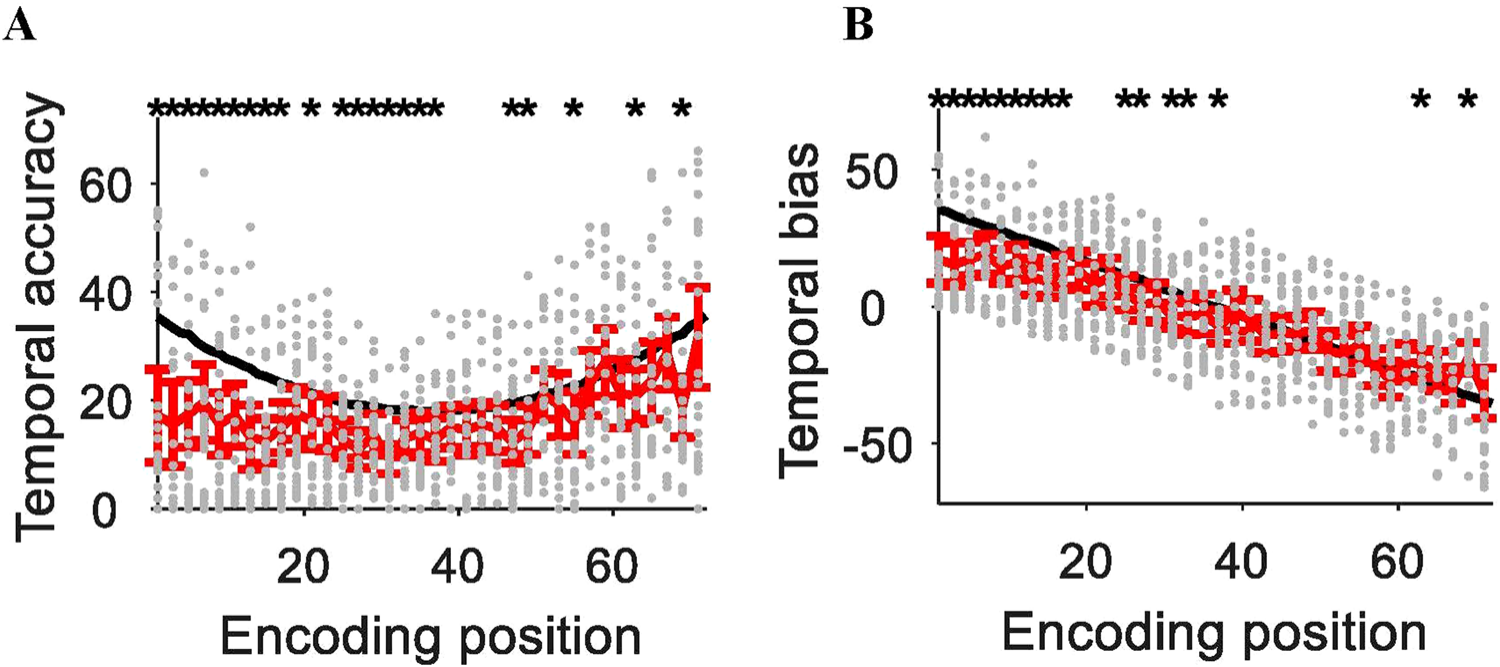
Timeline task performance for each encoding position in the sham condition for (A) temporal accuracy and (B) temporal bias. The higher the value in temporal accuracy, the more temporal error, that is, less accurate. The solid black curve represents the simulated chance level, the gray dots represent the data of each trial for each participant, and the red error bars represent the 95% confidence interval around subject-level mean performance. The asterisks represent significant difference between subject-level average performance and simulated chance performance (*p*< 0.05, uncorrected).

To compare empirical accuracy against chance level, we first subtracted empirical temporal accuracy from simulated temporal accuracy for each tested position in each participant, and then averaged the difference values to obtain temporal error relative to chance. One-sample t-tests showed that participants’ accuracy was significantly better compared to the simulated chance-level accuracy in each tACS condition (sham*: t*(23) = 11.48, 3 Hz:*t*(23) = 15.38, 8 Hz:*t*(23) = 12.50, all*ps*< 0.001, correctedα= 0.0167). We also compared the empirical temporal bias against simulated chance-level bias. The results showed that the empirical temporal bias was more negative than chance level in sham (*t*(23) = -4.49,*p*< 0.001) and 3 Hz (*t*(23) = -3.15,*p*= 0.005), but not in 8 Hz (*t*(23) = -1.85,*p*= 0.077; correctedα= 0.0167). The temporal accuracy and temporal bias of each trial for each participant and the differences between empirical error and chance level were presented in the Supplementary Material (Figs. S1, S2, and S3for sham, 3 Hz, and 8 Hz respectively).

#### 3.2.2 Temporal accuracy

**Fig. 5.**
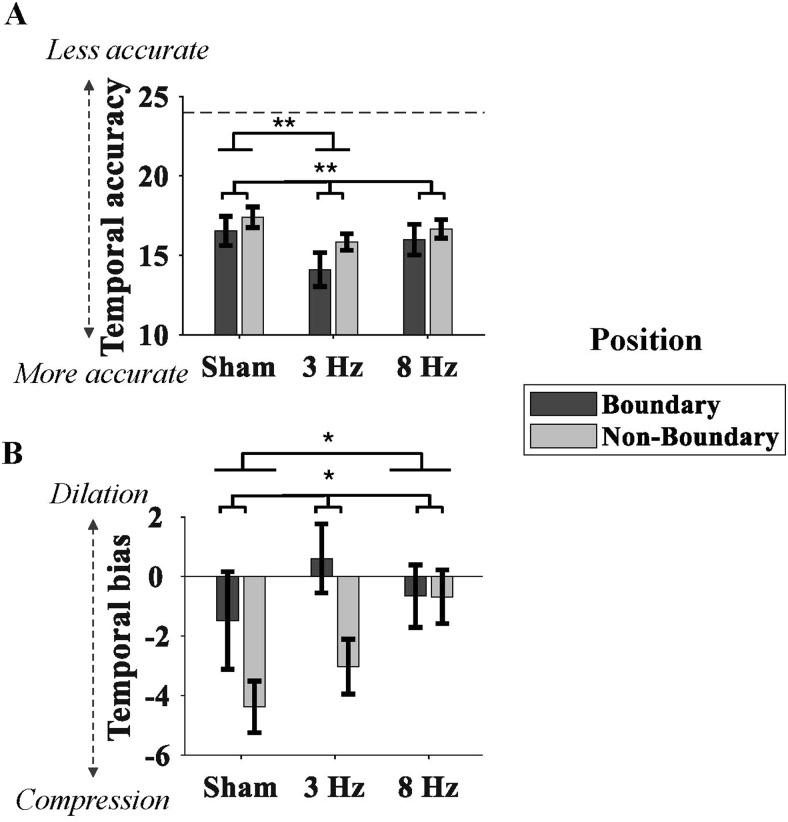
Results of the timeline task. (A) Absolute temporal error (temporal accuracy) as a function of tACS condition and encoding position. Dashed line represents chance-level accuracy of 24 (see main text). (B) Signed temporal error (temporal bias) as a function of tACS condition and encoding position. Error bars represent standard error of the mean. The asterisks indicate the uncorrected*p*value. *p< 0.05, **p< 0.01.

There were significant main effects of tACS (*F*(2, 46) = 3.53,*p*= 0.037,*η_p_^2^*= 0.13) and position (*F*(1, 23) = 8.37,*p*= 0.008,*η_p_^2^*= 0.27) on temporal memory accuracy (Fig. 5A), but there was no significant tACS x position interaction effect (*p*= 0.745). Pooled across positions, the slow theta stimulation significantly (correctedα= 0.0167) improved the temporal accuracy (mean [SE], 3 Hz = 15.00 [0.68]) compared to the sham condition (sham = 17.00 [0.64];*t*(23) = 3.17,*p*= 0.004, Cohen’s*d*= 0.62). The differences between fast theta and sham (*p*= 0.422) or fast and slow theta (*p*= 0.132) were not significant. In addition, pooled across tACS conditions, the temporal accuracy of the boundary item (i.e., the 1st item within a context, mean [SE] = 15.50 [0.63]) was significantly better than the non-boundary items (16.60 [0.40]), supporting the notion of a boundary effect of better temporal memory for boundary than non-boundary items. These findings suggested that slow theta stimulation improved temporal memory relative to sham condition and the precision of temporal memory of boundary item was superior to that of non-boundary item.

#### 3.2.3 Temporal bias

In the sham condition, the average temporal bias for each position was negative (Fig. 5BandSupplementary Fig. S4C), suggesting that participants consistently underestimated the temporal position of each item within a color context. This observation during the sham condition appears in line with an effect of temporal compression in memory (seeSection 4). A repeated-measures ANOVA revealed significant main effects of tACS (*F*(2, 46) = 3.45,*p*= 0.040,*η_p_^2^*= 0.13) and position (*F*(1, 23) = 7.65,*p*= 0.011,*η_p_^2^*= 0.25). The tACS x position interaction effect was not significant (*p*= 0.165). Pooled across positions, the temporal bias in the fast theta condition (8 Hz = -0.67 [0.72]) was reduced compared to the sham condition (sham = -2.93 [1.06];*t*(23) = 2.14,*p*= 0.043, Cohen’s*d*= 0.49), although this effect was significant at an uncorrected but not at correctedαthreshold. The temporal bias between slow theta and sham (*p*= 0.069) or slow and fast theta (*p*= 0.451) was not significant. The overall pattern of results was similar to the effect of tACS on temporal bias relative to chance performance in the above-described analysis. Pooled across tACS conditions, the temporal bias was smaller for the boundary position (-0.51 [0.97]) than non-boundary position (-2,70 [0.63]), indicating that participants were less likely to underestimate temporal position for the boundary item, compared to non-boundary items. In sum, fast theta tACS appeared to suppress a default tendency to underestimate temporal position during sham.

#### 3.2.4 Post-hoc verification using robust means

As shown inFigure 4, the distribution of temporal errors varied with serial position, with higher absolute errors for items presented near the beginning and end of the lists compared to items presented in the middle (see alsoSupplementary Materials). To verify that our results were not driven by outliers, we calculated the statistical models for temporal accuracy and bias using robust means, which are less sensitive to outliers. The results of these analyses supported our statistical conclusions of the raw means as presented above (seeSupplementary Materials, Fig. S5).

### 3.3 Relation between accuracy and bias after tACS

**Fig. 6.**
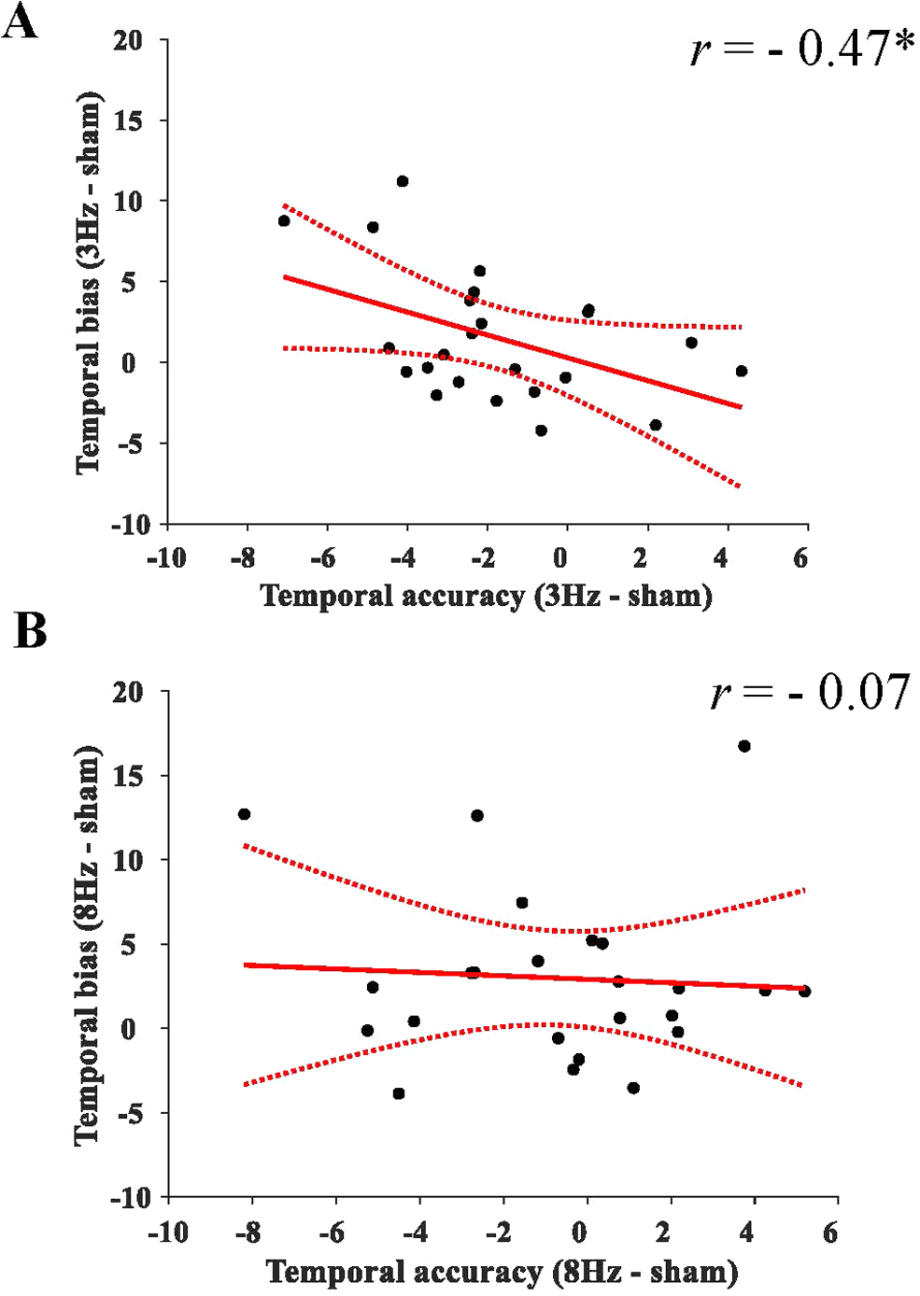
Correlations between tACS-induced changes (relative to sham) of temporal accuracy (abscissa) and temporal bias (ordinate) for slow (A) and fast theta stimulation (B). Solid line shows the ordinary least squares trend line and the dotted lines indicate +/- 95% confidence interval. *p< 0.05.

The findings thus far indicated that slow theta tACS improved temporal memory accuracy, while fast theta tACS diminished a temporal underestimation bias observed during sham. That is, the findings appeared to suggest a dissociation between slow and fast tACS and temporal accuracy vs. temporal bias. However, the non-significant interaction terms do not statistically support this notion. To explore the relationship between temporal bias and temporal accuracy, in a posthoc analysis we correlated the tACS-induced changes in temporal accuracy and bias for each of the two theta conditions (i.e., relative to sham stimulation). To this end, we first calculated the difference between slow theta tACS and sham for temporal accuracy, as well as for temporal bias, and then correlated the two difference metrics (Fig. 6). A negative difference value of temporal accuracy would indicate improved temporal accuracy for slow theta compared to sham. A negative difference value of temporal bias would indicate more temporal bias for slow theta relative to sham. Our analysis revealed a significant negative correlation between tACS-induced temporal bias and temporal accuracy for the effect of slow theta relative to sham (*r*= -0.47,*p*= 0.021;Fig. 6A). That is, tACS-induced enhanced temporal accuracy after slow theta stimulation was associated with reduced temporal bias. This finding appears in line with an overall improved temporal memory performance after slow theta stimulation relative to sham. However, no significant correlation was observed for fast theta (*r*= -0.07,*p*= 0.76;Fig. 6B), despite a significant tACS-induced reduction of temporal bias (as described above). The correlations differed significantly (*z *= -2.02,*p*= 0.044), which statistically supports the notion that slow theta stimulation affected temporal memory performance differently than fast theta stimulation.

### 3.4 Identifying sham vs. theta stimulation

Participants were asked to indicate which condition was related to sham or theta tACS at the end of the experiment. Analysis revealed that 12 out of 24 participants (50%) correctly identified the sham condition (chance level = 33%; sign rank test,*p*= 0.034). When taking identification of the sham condition statistically into account as a covariate in a repeated-measures ANOVA, the effects of the covariate were not significant either on the temporal bias (all*ps*> 0.49) or on the temporal accuracy (all*ps*> 0.33), suggesting that correct identification of the sham condition did not influence the effect of tACS on temporal position judgment.

### 3.5 The effect of tACS order

The order of tACS conditions was counterbalanced across participants. To statistically verify that tACS order did not affect our results, we included tACS order as a covariate in an expanded repeated-measures ANOVA for temporal accuracy and found no significant effect of tACS order (*p*= 0.51) or its interaction with tACS condition (*p*= 0.09) or with position (*p*= 0.48). Similarly, adding tACS order as a covariate for temporal bias did not reveal a significant effect of tACS order (*p*= 0.35) or its interaction with tACS condition (*p*= 0.40) or with position (*p*= 0.63). Therefore, we conclude that tACS order did not affect our results.

## 4 Discussion

This study represents the first investigation of the role of theta oscillations in temporal memory using non-invasive brain stimulation. The results showed that slow, but not fast, theta stimulation significantly improved temporal accuracy compared to the sham condition. These findings align with previous brain stimulation studies of non-temporal working memory performance (Bender et al., 2019;Wolinski et al., 2018), in which slow, but not fast theta tACS administered over fronto-parietal regions increased working memory capacity. These and our findings thus support the notion that slow theta oscillatory activity supports associative memory formation (Crespo-García et al., 2016;Kota et al., 2020;Lega et al., 2012;Lin et al., 2017). Furthermore, slow theta oscillations may not only play a role in memory processing but also serve as an associative glue that links distinct items during the encoding process and thereby enhance overall memory performance (Clouter et al., 2017;Gupta et al., 2012;Heusser et al., 2016). Our findings extend this notion of slow theta oscillatory activity to the formation of temporal memory.

Our temporal bias results were unexpected and require more explanation. The negative temporal bias in the sham condition indicated that, by default, participants rated temporal position earlier than the actual position in the timeline task. Further, the negative temporal bias was larger for non-boundary than for boundary positions, suggesting that contextual boundaries served as temporal anchors that diminish or otherwise protect against an inherent negative temporal bias that is pervasive to items presented away from those boundaries. These findings may be in line with previous observations of boundary-based temporal compression in event segmentation and temporal memory (Frisoni et al., 2021;Jeunehomme & D’Argembeau, 2020;Wen & Egner, 2022). For instance,Frisoni et al. (2021)explored temporal position memory judgments for movie clips taken from a cinematic feature, after participants either saw the full movie or only two-thirds of it. Results showed a larger negative temporal bias after having watched the incomplete movie, compared to the full movie. Further, the temporal bias increased for subsequently later clips in the movie. The authors reasoned that, for the incomplete movie, participants reconstructed an ending to the narrative in memory based on pre-knowledge of narrative movie schemas, which resulted in a relative compression of the actually observed clips in the movie’s timeline. In another study,Wen and Egner (2022)demonstrated boundary-based compression in a temporal distance judgment task. Like in our study, participants encoded sequences of images while contextual information changed regularly. Although the authors mainly described performance on a temporal order memory task, they also included a temporal distance task in which participants judged how temporally close a pair of test items was presented during the encoding phase. Results showed that participants underestimated temporal distance for test items drawn from the same context, while distance judgments were more accurate for test items presented close to contextual boundaries, compared to test items away from those boundaries. Our findings fit well to these results, and collectively support the notion that temporal compression is an inherent aspect of associative memory formation and segmentation.

In addition, we found that fast theta stimulation diminished temporal bias, compared to sham stimulation, but in the absence of a change in temporal accuracy, suggesting that fast theta stimulation affected the distribution of positive and negative errors without diminishing their magnitude. This effect could perhaps be alternatively explained by a manipulation of attentional processing, rather than of memory. Our choice of 8 Hz as the upper bound of the theta range overlaps with the lower bound of the alpha range (8–13 Hz), which has been amply associated to visual attentional processing (Hanslmayr et al., 2011;Jensen et al., 2012). Alpha tACS administered over the left superior parietal cortex has been shown to bias visual attentional processing to the left hemifield (Deng et al., 2019;Radecke et al., 2023;Schuhmann et al., 2019). When applied to our task, a timeline judgment could perhaps be considered a visuospatial task in disguise in which the temporal judgment is more akin to a line bisection judgment. In this scenario, left parietal alpha tACS would lead to timeline judgments biasing towards the left of the timeline. In fact, we found an opposite effect of a less leftward bias in the sense of a diminished negative temporal bias, compared to the more negatively biased (i.e., more leftward) judgments during sham.

Alternatively, our 8 Hz stimulation could have manipulated alpha power in the context of temporal perception and expectations. Several studies have shown decreased alpha power when a target is expected to appear in the next moment in time (Rohenkohl & Nobre, 2011;Samaha et al., 2015;van Diepen et al., 2015). Further, there is some evidence that faster alpha frequency may be related to a higher temporal resolution to separately perceive brief consecutive visual events in close temporal proximity (Samaha & Postle, 2015). In this light, our 8 Hz stimulation could have decreased temporal attentional resolution within the alpha range during encoding of the series of items, resulting in poorer temporal memory of those items. However, our 8 Hz stimulation did not significantly decrease performance relative to sham stimulation, suggesting that impaired temporal processing per se likely does not explain the effect of fast theta stimulation.

Instead, we suggest that the 8 Hz stimulation effect of diminished temporal bias points to a mnemonic origin, in which stimulation suppressed an*a priori*temporal compression bias. A recent study showed that peak frequency in the fast theta range in the hippocampus correlated with movement speed when human participants traversed through a virtual environment (Goyal et al., 2020), providing some evidence that fast theta oscillations may be related to temporal processing in the hippocampus. Alternatively, temporal bias may also be explained by theta-gamma coupling of consecutively phase-locked items in a theta cycle. Items phase-locked to a slow theta cycle would show a relatively smaller phase difference compared to phase-locking to a faster theta cycle. A smaller theta phase difference may lead to increased perceptual similarity of the phase-locked items (Ten Oever, Meierdierks, et al., 2020). This effect could translate to increased temporal similarity, that is, smaller temporal distance, and manifest as a negative temporal bias in our task. However, this notion contrasts with the suggestion of a functionally dissociable role of fast compared to slow theta in memory processing. The exact contribution of fast theta to memory formation and temporal processing remains unclear. Future studies will therefore have to elucidate how fast theta oscillations contribute to temporal memory performance.

Our findings may be related to a modulatory effect of theta tACS over parietal cortex on hippocampal function. There is growing evidence that transcranial electric stimulation of the parietal cortex affects hippocampal function, despite the low stimulation power at the cranium (Krause et al., 2019;Vieira et al., 2020;Wischnewski et al., 2023). We recently showed that theta tACS over parietal cortex modulates hippocampal-cortical connectivity more strongly than stimulation at higher frequencies (Kaiser et al., 2023). Although it is not possible to directly stimulate hippocampus with conventional non-invasive approaches, the existence of parietal-hippocampal connectivity makes it possible to influence hippocampal function indirectly by applying tACS to the parietal cortex. Further, the timeline task has been shown to activate the hippocampus during encoding, with more hippocampal activity for items that were subsequently recalled with more temporal accuracy (Jenkins & Ranganath, 2010;Montchal et al., 2019). Thus, it is reasonable to assume that the hippocampus plays a role in processing temporal position. Therefore, we suggest that future research includes monitoring the effects of theta tACS on hippocampal activity to confirm the correlation between scalp tACS and temporal memory.

It could be argued that our tACS results were caused by a non-neural origin. For example, electric stimulation over the scalp could trigger retinal phosphenes through volume conductance of the current (Schutter & Hortensius, 2010) or result in motor stimulation through transcutaneous peripheral nerve stimulation (Asamoah et al., 2019). We did not inquire if participants experienced tACS-induced phosphenes or peripheral events during stimulation, although none of the participants mentioned such experiences at debriefing. TACS may more reliably or strongly induce retinal phosphenes at beta or alpha stimulation frequencies (Kanai et al., 2008;Schwiedrzik, 2009) compared to theta frequencies, particularly when stimulating frontal and occipital areas (Evans et al., 2019;Kanai et al., 2010). We found no evidence of response time differences between the tACS conditions during encoding, suggesting that tACS-induced retinal or peripheral stimulation effects, if any, did not alter task performance. Alternatively, tACS-induced peripheral stimulation, if any, could also have affected post-stimulation memory performance. For example, sensory stimulation delivered at specific phases of endogenous oscillatory activity during encoding can enhance subsequent memory performance (Ngo et al., 2013;Ten Oever, De Weerd, et al., 2020). In this sense, tACS-induced phosphenes systematically triggered at relevant oscillatory phases could thereby enhance memory performance in our study. Our findings of differential effects of slow versus fast theta, in combination with above-mentioned considerations, are not consistent with this account. We therefore consider this scenario unlikely to explain our findings, although further research would be required to fully rule out non-neural stimulation effects.

The consideration of a few limitations is warranted. Firstly, we did not measure neural oscillations during the task or when participants received tACS stimulation. It would be insightful to explore whether the applied tACS frequencies aligned or interfered with the participants’ natural oscillatory patterns and how this alignment or interference may influence temporal memory, and provide an empirical test of the role of theta phase or power related to event segmentation and temporal memory formation (Griffiths & Fuentemilla, 2020;Jensen, 2006). Separating oscillatory stimulation signal from the EEG signal is notoriously difficult, but recent studies using amplitude-modulated tACS may provide a reliable way to remove the artifacts from tACS from EEG signal under certain circumstances (Haslacher et al., 2021,^2023^). Secondly, our participants were aware that they would be tested on their temporal memory of the list of items. Therefore, we cannot conclude if our results were due to active or implicit learning. Thirdly, conducting multiple sessions of tACS stimulation within 1 day might lead to potential after-effects of stimulation. The presence and duration of tACS after-effects is currently debated (Herrmann et al., 2013;Orendáčová & Kvašňák, 2021), with several studies showing limited evidence for post-stimulation oscillatory effects (Antal et al., 2008;Guerra et al., 2019;Nowak et al., 2017;Pozdniakov et al., 2021). We aimed to mitigate this risk using counterbalancing of tACS order across participants and found no statistical effect of counterbalancing on our results. We also used a distractor task to minimize potential after-effects on memory testing, but we cannot fully rule out potential after-effects of stimulation. Despite these limitations, this research marks an important step in unraveling the complex interplay between neural oscillations, cognitive functions, and temporal memory, opening avenues for future investigations in this intriguing field.

In conclusion, our study represents the first application of brain stimulation to test the contribution of theta stimulation on temporal memory, showing evidence for a functional dissociation in temporal memory formation of slow and fast theta.

## Data and Code Availability

The data and analysis code can be obtained from an Open Science Foundation page.https://osf.io/gf7e5/

## Author Contributions

Y.j.W. and V.V. designed the research. Y.j.W. and V. V. collected and analyzed the data and drafted the manuscript. P.D.W. and A.T.S. revised the manuscript and wrote the final version of the paper. A.T.S. provided brain stimulation materials. All authors approved the final version of the manuscript for submission.

## Funding

This experiment was partially financially supported by a grant from the China Scholarship Council awarded to Y.j.W. and V. V. (CSC 202108330031).

## Declaration of Competing Interest

The authors declare they have no conflict of interest.

## Acknowledgments

We thank Antonia Raissle for helping with developing the paradigm and Sofia Karageorgiou for helping with data collection. Felix Duecker and Sanne ten Oever provided comments on earlier versions of the manuscript.

## Supplementary Materials

Supplementary material for this article is available with the online version here:https://doi.org/10.1162/imag_a_00332.

## Supplementary Material

Supplementary Material
