# Supplementary material for: Distinct effects of slow and fast theta tACS in enhancing temporal memory
Source: Imaging Neurosci (Camb). 2024 Oct 24;2:imag-2-00332. doi: 10.1162/imag_a_00332 (PMC12290545; doi:10.1162/imag_a_00332)
Supplement: Supplementary Material [file imag_a_00332-supp.pdf]

Supplementary Materials

Distinct effects of slow and fast theta tACS in enhancing temporal memory

Yuejuan Wang, Peter De Weerd, Alexander T. Sack, Vincent van de Ven
yuejuan.wangpsych@gmail.com

Supplementary Results

Results separated for all four testing positions

In the following, we show the statistical results of the timeline task performance for all four testing positions. The repeated measures ANOVA included tACS (sham, 3Hz, 8Hz) and testing position (1st, 3rd, 5th, 7th) as within-subject factor. These results complement those described in the main manuscript, in which positions 3, 5 and 7 were pooled as "non-boundary position".

Response time. The main effects of tACS condition (p = 0.22) and encoding position (p = 0.377) as well as the interaction effect between them (p = 0.467) were not significant (Figure S4.A), suggesting that neither tACS nor the boundary affected the response time of temporal position judgements.

Temporal accuracy. There were significant main effects of tACS ($F(2, 46) = 3.68$, $p = 0.034$, $\eta_p^2 = 0.14$) and serial position ($F(3, 69) = 3.06$, $p = 0.034$, $\eta_p^2 = 0.12$) on temporal memory accuracy (Figure S4.B), but there was no significant tACS x position interaction effect (p = 0.271). Pooled across positions, the slow theta stimulation improved the temporal accuracy compared to the sham condition ($t(23) = 3.33$, p = 0.003, Cohen's $d = 0.63$). The differences between fast theta and sham (p = 0.314) or fast and slow theta (p = 0.165) were not significant. In addition, pooled across tACS conditions, the temporal accuracy of the boundary item (i.e., the 1st item within a context,) was significantly better than the 3rd ($t(23) = 3.67$, $p = 0.001$, Cohen's $d = 0.77$), but not significantly better than the 5th (p = 0.051) and 7th item (p = 0.282). There were no significant differences amongst the non-boundary items (3rd vs. 5th: p = 0.314; 3rd vs. 7th: p = 0.098; 5th vs. 7th: p = 0.539).

Temporal bias. A repeated measures ANOVA (Figure S4.C) revealed significant main effects of tACS ($F(2, 46) = 5.75$, $p = 0.006$, $\eta_p^2 = 0.20$) and position ($F(3, 69)$ =

3.13, p = 0.031, η_p^2 = 0.12). The tACS x position interaction effect was not significant (p = 0.299). Pooled across positions, the temporal bias in the fast theta condition was reduced compared to the sham condition (t(23) = 2.85, p = 0.009, Cohen's d = 0.74), while the temporal bias between slow theta and sham (p = 0.071) or slow and fast theta (p = 0.068) were not significant. Pooled across tACS conditions, the temporal bias was smaller for the boundary position than the 7th position (t(23) = 3.77, p < 0.001, Cohen's d = 0.72). Other comparisons between positions were not significant (1st vs. 3rd: p = 0.167; 1st vs. 5th: p = 0.079; 3rd vs. 5th: p = 0.929; 3rd vs. 7th: p = 0.183; 5th vs. 7th: p = 0.119).

Timeline statistical effects using robust means

We applied the robust mean of the temporal accuracy of temporal bias as a function of tACS condition (sham, 3 Hz, 8 Hz) and boundary (boundary, non-boundary). Robust means were calculated using Matlab routines. We extracted trials with response times within two standard deviations for each participant (inclusion of 95.22% of all trials).

Temporal accuracy. There were significant main effects of tACS (F(2, 46) = 4.51, p = 0.016, η_p^2 = 0.16) and boundary (F(2, 46) = 8.23, p = 0.009, η_p^2 = 0.26), but there was no significant tACS x boundary interaction effect (p = 0.69, Figure S5.A). The slow theta stimulation improved the temporal accuracy (mean [SE] 3 Hz = 16 [0.70]) compared to the sham condition (sham = 13.60 [0.74]; t(23) = 3.50, p = 0.002, *Cohen's d* = 0.68). The differences between fast theta and sham (p = 0.173) or fast and slow theta (p = 0.169) were not significant. In addition, the temporal accuracy of the boundary item (i.e., the 1st item within a context, mean [SE] = 14.00 [0.74]) was significantly better than non-boundary item (15.60 [0.44]).

Temporal bias. A repeated measures ANOVA revealed significant main effects of tACS (F(2, 46) = 4.41, p = 0.018, η_p^2 = 0.16) and boundary (F(2, 46) = 4.35, p = 0.048, η_p^2 = 0.16, Figure S5.B). The tACS x boundary interaction effect was not significant (p = 0.167). the temporal bias in the fast theta condition (8Hz = -0.30 [0.81]) was reduced compared to the sham condition (sham = -3.31 [1.06]; t(23) = 2.72, p = 0.012, *Cohen's d* = 0.65), while the temporal bias between slow theta and sham (p = 0.111) or slow and

fast theta (p = 0.171) were not significant. In addition, the temporal bias was smaller for boundary item (-0.65 [1.00]) than non-boundary item (-2.64 [0.64]).

Relation between accuracy and bias after tACS using robust means

We repeated the correlation analyses as reported in the main manuscript using robust means. The analysis revealed a significant negative correlation between temporal bias and temporal accuracy for the effect of slow theta relative to sham (r = -0.46, p = 0.022; Figure S6.A). That is, better temporal accuracy after slow theta stimulation was associated with less temporal bias. However, no significant correlation was observed for fast theta (r = 0.02, p = 0.92; Figure S6.B). The correlations differed significantly (z=-2.37, p=0.018), which statistically supports the notion that slow theta stimulation affected temporal memory performance differently than fast theta stimulation.

Supplementary Figures

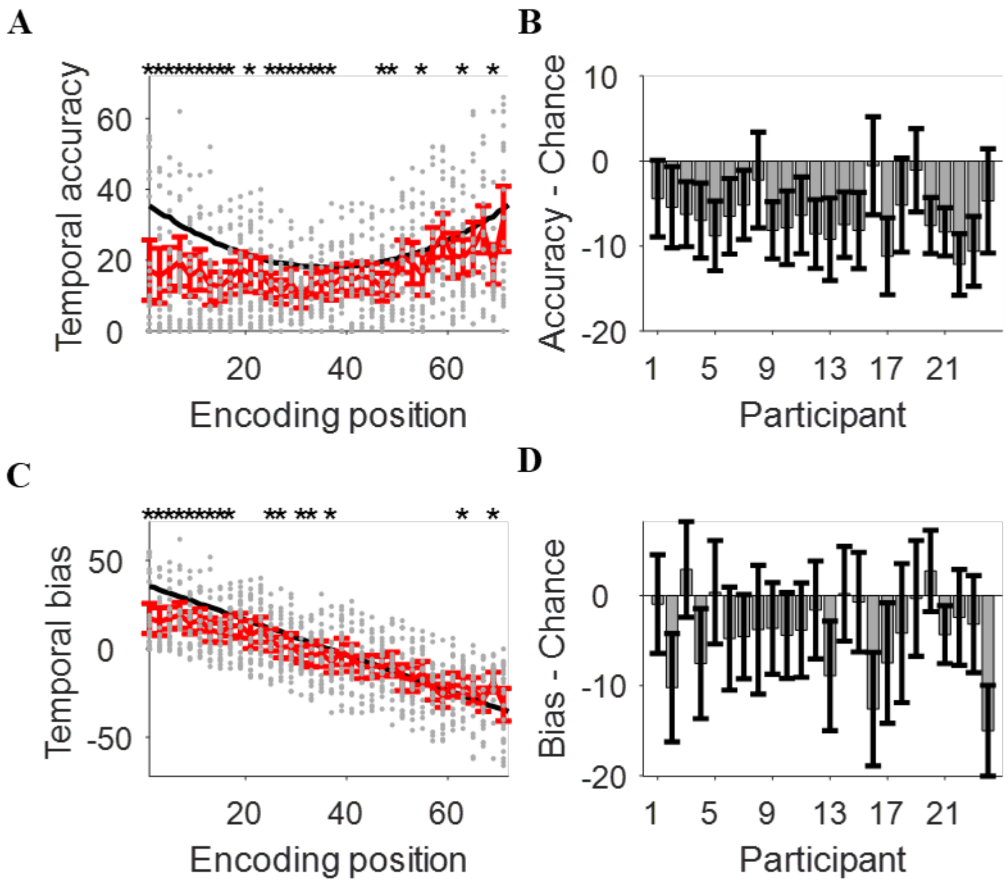

Figure S1. The temporal accuracy and temporal bias of each trial for each participant in the *sham* condition.

Temporal accuracy (A,B) and temporal bias (C,D) shown as a function of encoding position (A,C) and for each participant (B,D). The gray dots (A,C) represent the data of each trial for each participant and the red bars represent the confidence interval of 95% across participants. The solid black curves represent simulated chance-level performance (10,000 iterations of shuffled position indexes). The asterisks represent the difference between chance level and temporal accuracy is significant ($p < 0.05$). B and D show the difference between temporal accuracy (B) or temporal bias (D) and chance level (calculated for each encoding position) averaged across trials for each participant. The black bars represent the 95% confidence interval across trials for each participant. Panels A and C were also shown in the main manuscript.

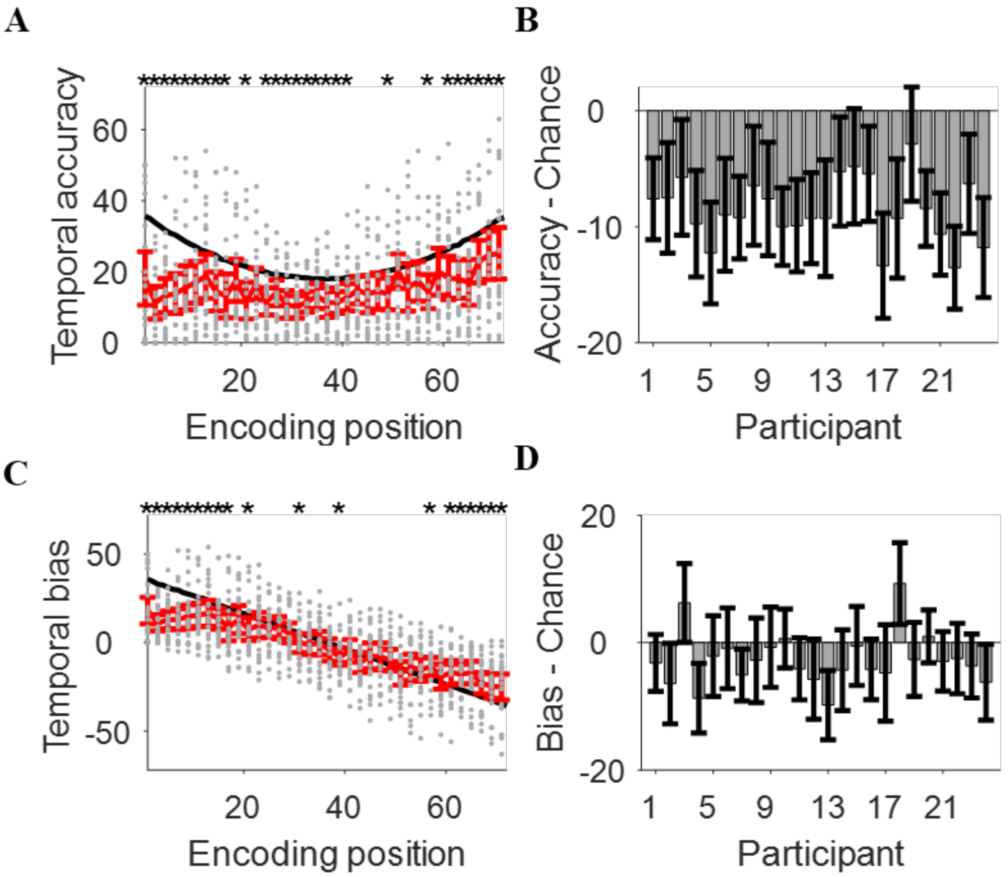

Figure S2. The temporal accuracy and temporal bias of each trial for each participant in *3 Hz* condition. See caption of Figure S1 for an explanation of figure formats.

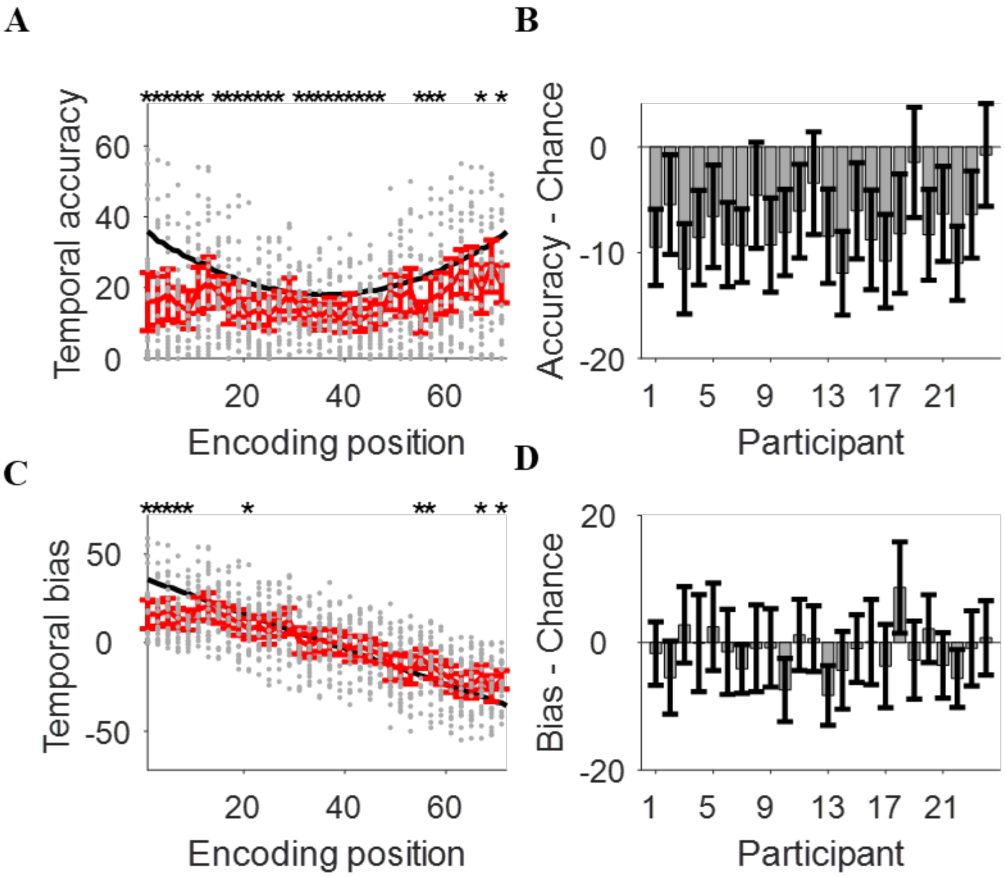

Figure S3. The temporal accuracy and temporal bias of each trial for each participant in *8 Hz* condition. See caption of Figure S1 for an explanation of figure formats.

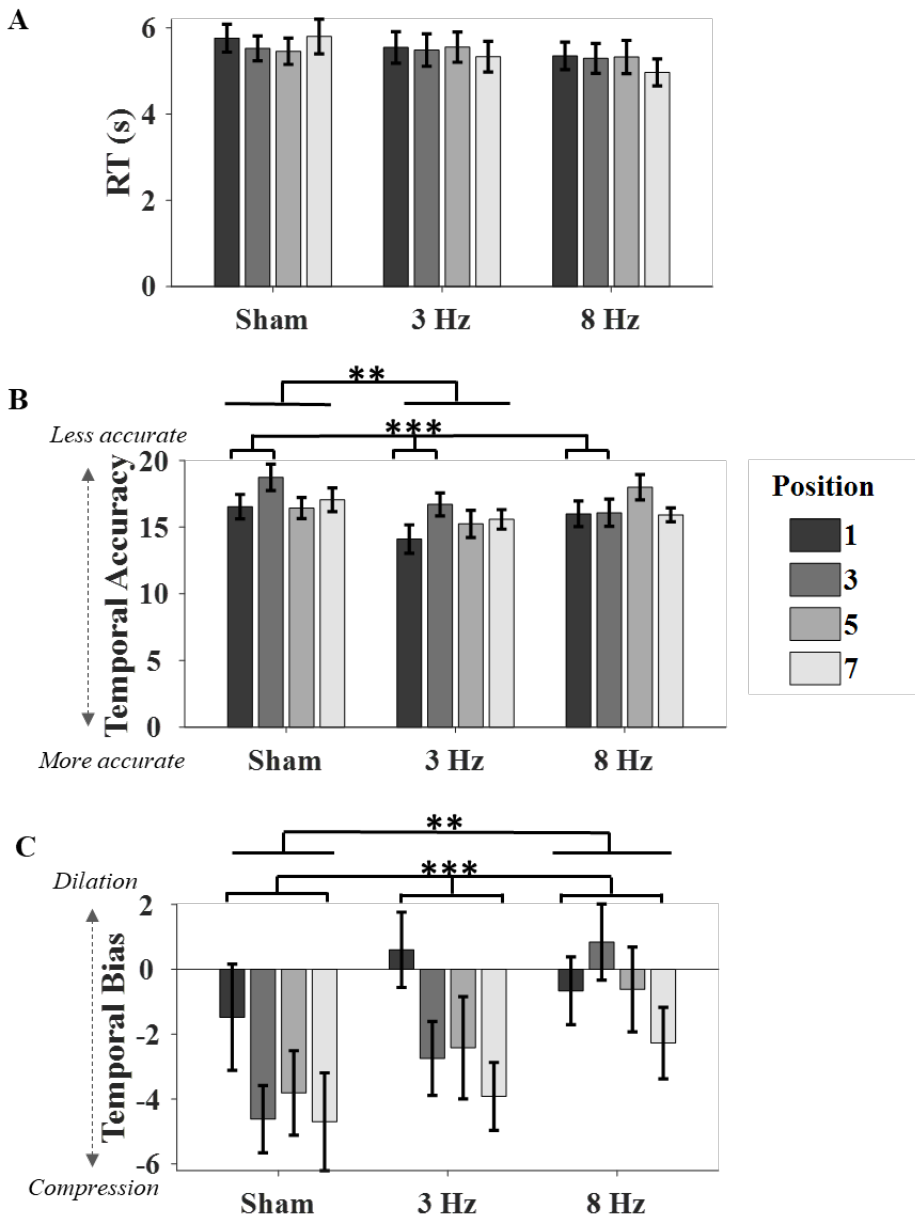

Figure S4. **Timeline task results separated for four testing positions.** (A) Response time of the temporal position task as a function of tACS condition and encoding position. (B) Absolute temporal error / temporal accuracy as a function of tACS condition and encoding position. (C) Temporal error / temporal bias as a function of tACS condition and encoding position. Error bars represent standard error of the mean. ** $p < 0.01$, *** $p < 0.001$.

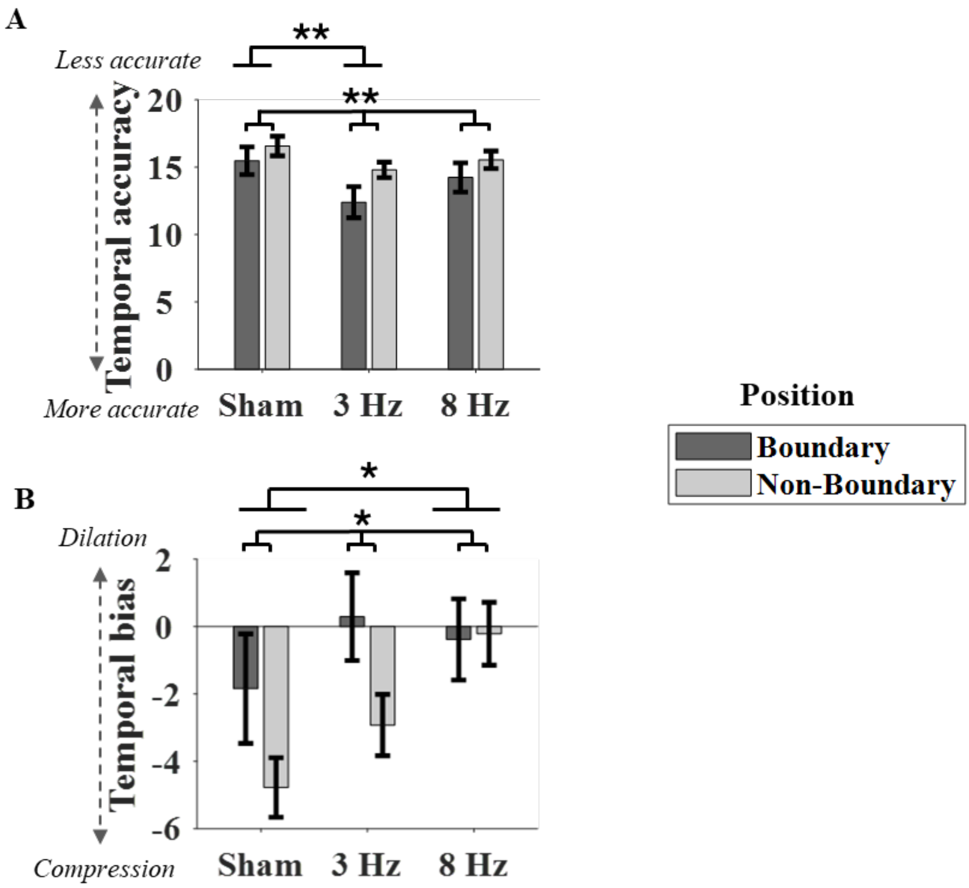

Figure S5. Timeline task results using robust means. (A) Absolute temporal error / temporal accuracy as a function of tACS condition and encoding position. (B) Temporal error / temporal bias as a function of tACS condition and encoding position. Error bars represent standard error of the mean. * $p < 0.05$, ** $p < 0.01$.

A

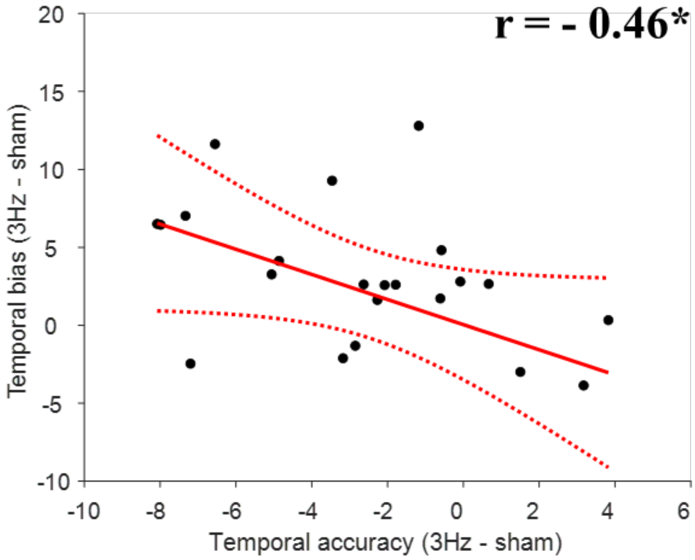

B

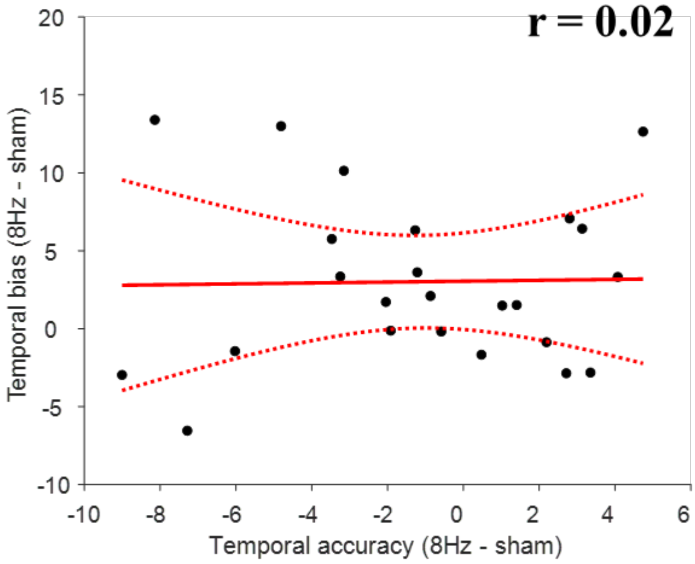

Figure S6. Correlations based on robust means. Correlations between tACS-induced changes (relative to sham) of temporal accuracy (abscissa) and temporal bias (ordinate) for slow (A) and fast theta stimulation (B). Solid line shows the ordinary least squares trend line and the dotted lines indicate +/- 95% confidence interval. * $p < 0.05$.
